# Supplementary material for: Anatomical and Transcriptome Analyses of Moso Bamboo Culm Neck Growth: Unveiling Key Insights
Source: Plants (Basel). 2023 Oct 4;12(19):3478. doi: 10.3390/plants12193478 (PMC10574802; doi:10.3390/plants12193478)
Supplement: Supplementary file 1 [file plants-12-03478-s001.zip › plants-2597068-supplementary.pdf]

Table S1 Summary of subread sequences

Sample	Subreads base(G)	Subreads number	Average subreads length	N50
CulmNeck	16.68	15229362	1096	2137

Table S2 Summary of circular consensus sequences (CCS)

Sample	CCS_number	Min_length	Max_length	Mean_length	N50
CulmNeck	441914	50	14937	2000	3360

Table S3 Summary of full-length non-chimericreads (FLNC)

Sample	FLNC_number	Min_length	Max_length	Mean_length	N50
CulmNeck	249071	200	14342	2444	3505

Table S4 Summary of polished consensus sequences

Sample	Consensus_number	Min_length	Max_length	Mean_length	N50
CulmNeck	161160	131	14342	2373	3510

Table S5 Summary of transcripts after the correction by Illumina sequencing data

Sample	Type	Total_nucleotides	Total_number	Mean_length	Min_length	Max_length	N50	N90
CulmNeck	Before_correct	382388265	161160	2373	131	14342	3510	1526
CulmNeck	After_correct	382239200	161160	2372	131	14335	3507	1526

Table S6 Summary of GMAP alignment

Sample name	Total reads	Total mapped	Unmapped	Multiple mapped	Uniquely mapped	Reads map to '+'	Reads map to '-'
CulmNeck	161160	148595(92.2%)	12565(7.8%)	17246(10.7%)	131349(81.5%)	76092(47.22%)	55257(34.29%)

Table S7 Summary of isoforms

Samplename	Isoforms number	Isoforms of Known genes	Novel isoform of known genes	Isoforms of novel genes	Isoform mean length	N50
CulmNeck	73167	8676	56213	8278	2370.52	3488

Table S8 Summary of Illumina sequencing data

Sample name	Raw reads	Clean reads	Clean bases	Error rate(%)	Q20(%)	Q30(%)
CNS3_1	63146390	60135930	9.02G	0.03	98.01	94.20
CNS3_2	61812660	60152616	9.02G	0.03	97.85	94.09
CNS3_3	52234232	51243152	7.69G	0.02	97.95	94.31
CNS3_4	53147914	51505730	7.73G	0.03	97.85	94.20
CNS4_1	50927388	49547882	7.43G	0.03	97.94	94.27
CNS4_2	53158240	45776424	6.87G	0.03	97.61	93.72
CNS4_3	53055588	50971604	7.65G	0.03	97.48	93.64
CNS4_4	53666494	51621084	7.74G	0.03	97.92	94.25
CNS4_5	57090332	54371648	8.16G	0.02	97.93	94.33
CNS5_2	47839034	46981680	7.05G	0.03	97.74	93.79
CNS5_3	51975456	50767920	7.62G	0.02	98.08	94.45
CNS5_4	49071914	47822446	7.17G	0.03	97.62	93.67
CNS5_5	64494248	63273080	9.49G	0.03	97.71	93.92

Table S9 The primer sequences for qRT-PCR

Gene Name	Sequences (5'–3')
TIP41-S	GAGGATACTGATGACCAGATTGACC
TIP41-A	CACTCGCACTGTAAGGAATGAAATA
RSM1-S	TAGCTTCTTCTCGAGCCTCA
RSM1-A	TCGTAGTAGCGCTTCACCTC
RSM3-S	GATATAATTGGATGGCGTCG
RSM3-A	TCGTAGTAGCGCTTCACCTC
MYBH-S	CCACAGAGGCCTAGAGAACA
MYBH-A	CAAGTGAAGACGAGGACGAC
MYB70-S	TCAGAGCAGCCGAAGAATAA
MYB70-A	ATCAACCGACGCAAACCTAC

TIP41: *tonoplast intrinsic protein 41;* ***RSM***: *Radialis-like SANT/MYB;* ***MYBH:*** *MYB Hypocotyl elongation-related.*
